# Whole-Genome Sequence Analysis of CTX-M Containing *Escherichia coli* Isolates from Retail Meats and Cattle in the United States

**DOI:** 10.1089/mdr.2018.0206

**Published:** 2018-09-10

**Authors:** Daniel A. Tadesse, Cong Li, Sampa Mukherjee, Chih-Hao Hsu, Sonya Bodeis Jones, Stuart A. Gaines, Claudine Kabera, Guy H. Loneragan, Mary Torrence, Dayna M. Harhay, Patrick F. McDermott, Shaohua Zhao

**Affiliations:** ^1^ Division of Animal and Food Microbiology, U.S. FDA, CVM, Laurel, Maryland.; ^2^ Texas Tech University, Department of Animal and Food Science, Lubbock, Texas.; ^3^ U.S. FDA–CFSAN, Office of Applied Research and Safety Assessment (OARSA), Laurel, Maryland.; ^4^ USDA–ARS, U.S. Meat Animal Research Center, Meat Safety and Quality Research Unit, Clay Center, Nebraska.

**Keywords:** *Escherichia coli*, ESBL, CTX-M

## Abstract

In recent years, there have been increased reports on the detection of extended-spectrum beta-lactamase (ESBL)-producing *Escherichia coli* and *Salmonella* strains from food-producing animals and animal products in the United States. We characterized 18 ESBL *E. coli* isolates from cattle (*n* = 5), chicken breast (*n* = 5), ground turkey (*n* = 6), ground beef (*n* = 1), and pork chops (*n* = 1) that were collected by the National Antimicrobial Resistance Monitoring System (NARMS) between 2011 and 2015. *In vitro* antimicrobial susceptibility testing was done against a panel of 14 antimicrobials followed by a secondary panel of 9 β-lactam agents. Whole-genome sequencing was used to characterize the resistome, plasmids, and the genetic structures of the ESBL genes. All ESBL-producing *E. coli* isolates were resistant to at least three antimicrobial classes and carried various *bla*_CTX-M_ genes. Most of the cattle and ground turkey isolates carried *bla*_CTX-M-27_. In chicken breast isolates, *bla*_CTX-M-1_ was present as part of an IS*Ecp1* transposition unit carried on a plasmid that shares sequence similarity with the backbone structure of the IncI plasmid. Isolates carrying the *bla*_CTX-M-14_ and *bla*_CTX-M-15_ genes, widely distributed in human clinical isolates, were also isolated. To our knowledge, this is the first report of the widely distributed *bla*_CTX-M-14_ and *bla*_CTX-M-15_ in *E. coli* isolates from retail meat samples in the United States. Different insertional sequences were identified upstream of these *bla*_CTX-Ms_, including IS*Ecp1*, IS*26*, and IS*903-D*. CTX-M in *E. coli* from food animals and retail chicken breast were often present on plasmids with other resistance genes. Other resistance genes identified included *aadA*, *strA*, *strB*, *aac(3)-IId*, *aac(3)-VIa*, *aph(3′)-Ic*, *bla_TEM_*, *bla_HERA-3_*, *floR*, *sul1*, *sul2*, *catA1*, *tetA, tetB*, *dfrA*, and *qacE*. These data describe the emergence of CTX-M-carrying *E. coli* isolates in food animals and animal products monitored by NARMS program.

## Introduction

Extended-spectrum beta-lactamases (ESBLs) are the most common cephalosporin resistance mechanism reported in members of the Enterobacteriaceae family.^1^ ESBLs are a group of enzymes with the ability to hydrolyze oxyimino-cephalosporins and thus cause resistance to cefotaxime, ceftazidime, ceftriaxone, cefuroxime, and cefepime, as well as monobactams (*e*.*g*., aztreonam).^2^ The introduction of extended-spectrum cephalosporins in clinical practice in the 1970s was soon followed by reports of resistant strains of Enterobacteriaceae producing ESBLs.^3^ Since then, the occurrence of infection due to ESBL-resistant Enterobacteriaceae has rapidly increased and has become a major problem worldwide. In the United States, the Centers for Disease Control and Prevention estimates 26,000 infections and 1,700 deaths annually due to ESBL-producing Enterobacteriaceae.^4^

CTX-M-producing *Escherichia coli* have been associated with both hospital-acquired and community infections, mostly in urinary tract infections and bacteremia.^5–8^ Studies over the last decade have shown that CTX-M-type ESBLs have become the predominant enzyme type in many parts of the world,^9^ and have spread rapidly through clinical populations of Enterobacteriaceae.^10^ Reports from several countries describe the presence of CTX-M-producing *E. coli* strains in apparently healthy food animals^11–13^ and food animal products,^12^ as well as pets^14^ and wild birds.^15^ Although CTX-M-producing strains appear to have quickly spread worldwide, the increased prevalence of *E. coli* carrying these β-lactamases in numerous U.S. hospitals became apparent in the early 2000s.^7,10,16^

Intestinal carriage of CTX-M-producing bacteria in food-producing animals and contamination of retail meat might contribute to increased occurrences of infections with ESBL-producing bacteria in humans. A study on the presence of indistinguishable *E. coli* genotypes carrying CTX-M genes obtained from poultry, poultry products, and human clinical samples in the Netherlands has suggested the possible exchange of these genes through the food chain.^12^ In the United States, few CTX-M ESBLs have been reported from food animals and animal products.^11,17–19^ The National Antimicrobial Resistance Monitoring System (NARMS) monitors changes in antimicrobial susceptibilities of zoonotic foodborne bacteria to medically important antimicrobials, including β-lactam antibiotics. Whole-genome sequencing has improved our ability to monitor resistomes and helps to identify and characterize emerging resistance genes and mobile genetic elements that facilitate the spread of these genes. The aim of this study was to investigate and characterize antimicrobial resistance genes and mobile genetic elements associated with phenotypically positive ESBL *E. coli* isolates recovered from cattle and retail meat samples collected through the NARMS program between 2011 and 2015. This information will help to characterize the molecular epidemiology of CTX-M carrying *E. coli* isolates in food animals and animal products monitored by the NARMS program.

## Materials and Methods

### Bacterial strains

Eighteen phenotypically positive ESBL *E. coli* isolates recovered from cattle fecal samples (*n* = 5) and retail meats (chicken breast [*n* = 5], ground turkey [*n* = 6], ground beef [*n* = 1], and pork chops [*n* = 1]) by the NARMS program between 2011 and 2015 were identified and selected for characterization. The isolates were identified from a total of 8,721 *E. coli* isolates recovered from fecal samples of healthy cattle and retail meat samples. The fecal isolates (*n* = 3,079) were recovered from healthy cattle as part of a NARMS on-farm pilot program to monitor antimicrobial resistance in foodborne pathogens. The retail meat *E. coli* isolates (*n* = 5,642) were recovered from chicken breast, chicken wing, pork chops, ground beef, and ground turkey.

### *In vitro* antimicrobial susceptibility testing

The bacterial isolates were tested for antimicrobial drug susceptibility using the Sensititre™ semiautomated antimicrobial susceptibility system (ThermoFisher Scientific, Trek Diagnositics, Cleveland, OH) following the manufacturer's instructions. The antimicrobials tested were as follows: amoxicillin/clavulanic acid (AUG), ampicillin (AMP), azithromycin (AZM), cefoxitin (FOX), ceftiofur (TIO), ceftriaxone (CRO), chloramphenicol (CHL), ciprofloxacin (CIP), gentamicin (GEN), nalidixic acid (NAL), streptomycin (STR), sulfamethoxazole (SMX), tetracycline (TET), and trimethoprim/sulfamethoxazole (SXT).

A total 321 *E. coli* isolates with minimum inhibitory concentrations (MICs) ≥8 μg/mL for ceftiofur and/or ≥4 μg/mL for ceftriaxone for isolates recovered before 2015 and ≥2 μg/mL for isolates recovered in 2015 were selected for further testing with a second panel of 9 β-lactam antimicrobials: aztreonam (ATM), cefquinome (CQN), imipenem (IMI), cefepime (FEP), piperacillin–tazobactam (TZP), ceftazidime (TAZ), ceftazidime–clavulanic acid (CAZ/CLA), cefotaxime (FOT), and cefotaxime–clavulanic acid (CTX/CLA). The Clinical and Laboratory Standards Institute (CLSI) confirmatory test for ESBL production was used and is based on cefotaxime and ceftazidime MICs with and without clavulanic acid. Isolates showing a three or more twofold concentration decrease in the cefotaxime and ceftazidime MICs when tested in combination with clavulanate versus the MICs of cefotaxime and ceftazidime when tested alone are considered ESBL^+^.^20^

*E. coli* ATCC 25922, *Enterococcus faecalis* ATCC 29212, *Staphylococcus aureus* ATCC 29213, *Pseudomonas aeruginosa* ATCC 27853, and *Klebsiella pneumoniae* ATCC 7000603 were used as quality control organisms for MIC determinations. Results were interpreted according to CLSI guidelines for broth microdilution methods with the exception of STR (NARMS resistance breakpoint, ≥32 μg/mL), AZM (NARMS resistance breakpoint, ≥32 μg/mL), and CQN (NARMS resistance breakpoint, ≥32 μg/mL).^20^

### Conjugation

Conjugation experiments using a plate mating protocol were used to determine the transferability of resistance phenotypes and localize CTX-M genes to conjugative plasmids. We selected seven *E. coli* isolates that carried different CTX-M genes [N36254PS (*bla*_CTX-M-32_), N36410PS (*bla*_CTX-M-27_), N37058PS (*bla*_CTX-M-32_), N40513 (*bla*_CTX-M-1_), N40607 (*bla*_CTX-M-1_), N46045 (*bla*_CTX-M-15_), and N51980 (*bla*_CTX-M-14_)] as donor cells. MAX Efficiency^®^ DH5α™ *E. coli* Competent Cells (Invitrogen, Carlsbad, CA) were used as recipients. The donors and recipients were grown in 2 mL LB medium (Becton Dickinson, Sparks, MD) at 37°C in a shaker incubator for 16–18 hours. Ten microliters of donor cells were spotted on top of 10 μL of recipient strain (DH5α) on blood agar plates and incubated at 37°C overnight. Each co-culture was then scraped from the plate and resuspended in 1 mL LB broth. Ceftiofur and nalidixic acid (Sigma-Aldrich, St. Louis, MO) were used as selective agents for the donor and recipient strains, respectively. Transconjugants were selected on LB agar containing nalidixic acid (30 μg/mL) and ceftiofur (4 μg/mL). The MICs of donors, recipients, and transconjugants were determined using the Sensititre semiautomated antimicrobial susceptibility system. The β-lactam susceptibility testing panel was used to confirm the phenotype.

Five transconjugants from each experiment were tested for the presence of CTX-M genes using PCR primers designed for CTX-M-1 group (forward primer, 5′-ATGGTTAAAAAATCACTGCGTCAGT-3′; reverse primer, 5′-TTACAAACCGTTGGTGAGATTTTAGCC-3′) and CTX-M-9 group (forward primer, 5′-ATGGTGACAAAGAGAGTGCAACGG-3′; reverse primer, 5′-TTACAGCCCTTCGGCGATGATTCT-3′). The expected amplicon fragment size for CTX-M group 1 and 9 was 876 and 846 bp, respectively. The PCR amplification conditions included initial denaturation at 95°C for 10 minutes, 30 cycles of denaturing at 94°C for 30 seconds, annealing at 58°C for 60 seconds, and extension at 72°C for 60 seconds, and followed by final extension at 72°C for 7 minutes.

### PCR-based plasmid replicon typing of transconjugants

Genomic DNA was extracted using the DNeasy Blood and Tissue kit (Qiagen, Valencia, CA) following the manufacturer's instructions. Amplification of plasmid replicon targets was carried out following the protocol described by Johnson *et al.*^21^ with minor modifications for IncP characterization. For IncP, a simplex PCR with an annealing temperature of 65°C was used. The amplified products were separated by gel electrophoresis on 1.0% agarose gels.

### Whole-genome sequencing

Whole-genome sequencing was used to characterize the resistome and plasmids in all strains (*n* = 18). Briefly, DNA was extracted using the DNeasy Blood and Tissue Kit (Qiagen) following the manufacturer's instructions. Whole-genome sequencing was performed on the MiSeq Desktop Sequencer using v2 sequencing reagent kits (Illumina, San Diego, CA). A *de novo* assembly was performed using CLC Genomics Workbench version 8.0 (Qiagen). Contigs of less than 200 bp were removed from analysis. The number of assembled contigs ranged between 78 and 258 with an average coverage of 50 × .

### Resistome analysis

Resistance genes were identified using BLASTX^a^ and the ResFinder resistance gene database.^22^ The BLASTX results were processed with in-house PERL scripts to identify antimicrobial resistance genes using an 85% amino acid identity and 50% minimum sequence length.

### Phylogenetic analysis

The Center for Food Safety and Applied Nutrition (CFSAN) SNP pipeline^b^ was used to create the single nucleotide polymorphism (SNP) matrices from sequence data for the phylogenetic analysis. SNP redundancy by linkage disequilibrium was reduced and the phylogenetic tree was constructed with the maximum likelihood algorithm using the SNPhylo package.^23^

### Plasmid profiling

Identification of the plasmid type was done using the PlasmidFinder database.^c^ The cutoff threshold for identity was set at 95% to determine the existence for a particular plasmid.

### Multilocus sequence typing profiling

*E. coli* multilocus sequence typing (MLST) allelic profiles and sequences were downloaded from the PubMLST database.^d^ A total of 7,113 profiles for 7 different loci were used for the MLST. The SRST2 pipeline^24^ was used to determine the MLST type for our *E. coli* isolates.

### Nucleotide sequence accession numbers

The whole-genome sequence data reported in this study have been deposited at DDBJ/ENA/GenBank under the following accession numbers: AZCE00000000, AZCG00000000, AZCB00000000, AZCC00000000, AZCD00000000, AZCF00000000, AZCH00000000, NTMS00000000, NTMT00000000, NTMU00000000, NTMV00000000, NTMW00000000, NTMX00000000, NTMY00000000, NTMZ00000000, NTNA00000000, NTNB00000000, and NTNC00000000.

## Results

### *In vitro* antimicrobial susceptibility testing of *E. coli* isolates

The antimicrobial resistance profiles of phenotypically positive ESBL *E. coli* isolates are shown in Table 1. All the isolates were resistant to ampicillin, ceftiofur, ceftriaxone, and cefotaxime, as expected. Three of the 5 cattle and 2 of the 13 retail meat *E. coli* isolates were resistant to cefquinome, a fourth-generation cephalosporin. In addition, one of the cattle *E. coli* isolates and two of retail meat isolates showed resistance to aztreonam, a monobactam. Among the 18 strains producing ESBL, all had a three or more twofold concentration decrease in MIC for cefotaxime and ceftazidime in combination with clavulanic acid than the MIC when tested alone (Table 2). Other non-β-lactam resistances observed were to sulfisoxazole (15/18), tetracycline (14/18), chloramphenicol (3/18) streptomycin (9/18), nalidixic acid (1/18), and trimethoprim/sulfamethoxazole (4/18).

**Table 1.** Minimum Inhibitory Concentration Values of Extended-Spectrum Beta-Lactamase-Positive *Escherichia coli* Isolates Obtained from Fecal Samples of Healthy Cattle and Retail Meat Samples

		*Antimicrobials*^a^													
*ID*	*Source*	*AMC ≥32/16*	*AMP ≥32*	*AZM*^b^*≥32*	*FOX ≥32*	*CRO ≥4*	*MEM ≥4*	*CHL ≥32*	*CIP ≥4*	*NAL ≥32*	*GEN ≥16*	*STR*^b*≥32*^	*TET ≥16*	*FIS ≥16*	*SXT ≥4/76*
N36254PS	Farm, fecal	4	>32	4	8	8	≤0.06	8	≤0.015	2	0.5	32	>32	>256	≤0.12
N36410PS	Farm, fecal	8	>32	4	8	>64	≤0.06	8	≤0.015	2	0.5	16	≤4	>256	>4
N37058PS	Farm, fecal	4	>32	4	4	32	≤0.06	>32	1	4	0.5	32	>32	>256	0.25
N37122PS	Farm, fecal	4	>32	4	8	>64	≤0.06	8	≤0.015	2	1	8	≤4	>256	>4
N37139PS	Farm, fecal	4	>32	4	8	64	≤0.06	8	≤0.015	2	0.5	8	≤4	>256	>4
N40513	Chicken breast	8	>32	4	4	64	≤0.06	4	≤0.015	2	0.5	8	>32	>256	0.5
N40607	Chicken breast	8	>32	2	8	>64	≤0.06	4	≤0.015	4	0.5	16	>32	>256	0.5
N43684	Chicken breast	4	>32	4	4	>64	≤0.06	4	≤0.015	2	0.5	8	≤4	>256	1
N44807	Ground turkey	8	>32	4	8	>64	≤0.06	8	≤0.015	4	≤0.25	>64	>32	≤16	≤0.12
N46045	Ground beef	32	>32	4	8	>64	≤0.06	>32	≤0.015	4	0.5	>64	>32	>256	>4
N51980	Pork chop	8	>32	4	4	64	≤0.06	8	≤0.015	2	0.5	>64	>32	≤16	≤0.12
N53976	Ground turkey	4	>32	2	4	32	0.25	4	0.5	2	4	8	>32	>256	2
N56041	Ground turkey	16	>32	2	4	>64	≤0.06	>32	0.12	>32	0.5	>64	32	>256	≤0.12
N56738	Ground turkey	8	>32	4	4	>64	≤0.06	8	≤0.015	2	1	64	>32	>256	≤0.12
N58201	Chicken breast	8	>32	4	4	64	≤0.06	4	≤0.015	4	4	8	>32	>256	0.5
N60559	Chicken breast	8	>32	4	8	64	≤0.06	4	≤0.015	2	>16	4	>32	>256	1
N60592	Ground turkey	8	>32	4	4	64	≤0.06	4	≤0.015	2	16	64	>32	>256	≤0.12
N63148	Ground turkey	4	>32	4	2	64	≤0.06	8	≤0.015	2	0.5	8	32	≤16	≤0.12

^a^ CLSI breakpoint.

^b^ NARMS breakpoint.

AMC, amoxicillin/clavulanic acid; AMP, ampicillin; AZM, azithromycin; CHL, chloramphenicol; CIP, ciprofloxacin; CRO, ceftriaxone; FIS, sulfisoxazole; FOX, cefoxitin; GEN, gentamicin; MEM, meropenem; NAL, nalidixic acid; STR, streptomycin; SXT, trimethoprim/sulfamethoxazole; TET, tetracycline.

**Table 2.** Minimum Inhibitory Concentration Values from the Extended-Spectrum Beta-Lactamase Panel for *Escherichia coli* Isolates Obtained from Fecal Samples of Healthy Cattle and Retail Meat Samples with Diminished Susceptibility or Resistance to Broad-Spectrum Cephalosporins

		*Antimicrobials*^a^								
	*Source*	*ATM ≥16*	*FEP ≥16*	*CTX ≥4*	*CQN*^b^*≥32*	*CAZ ≥16*	*IPM ≥4*	*TZP ≥128/4*	*CTX/CLA —*	*CAZ/CLA —*
N36254PS	Farm, fecal	2	1	8	4	1	0.12	≤0.5	≤0.06	≤0.06
N36410PS	Farm, fecal	16	4	64	>32	4	0.12	8	0.25	0.12
N37058PS	Farm, fecal	8	4	32	16	4	0.25	1	≤0.06	0.12
N37122PS	Farm, fecal	8	8	128	32	2	0.12	1	≤0.06	0.12
N37139PS	Farm, fecal	8	4	64	32	8	0.12	≤0.5	≤0.06	≤0.06
N40513	Chicken breast	8	4	16	8	1	0.25	1	≤0.06	0.25
N40607	Chicken breast	16	8	64	16	8	0.25	2	≤0.06	0.25
N43684	Chicken breast	>32	16	128	>32	8	0.25	2	0.12	0.25
N44807	Ground turkey	8	4	64	16	1	0.25	2	0.12	0.25
N46045	Ground beef	4	8	32	32	1	0.12	1	≤0.06	0.25
N51980	Pork chop	4	2	32	8	4	0.12	2	≤0.06	0.12
N53976	Ground turkey	8	8	32	16	1	0.25	2	≤0.06	0.12
N56041	Ground turkey	4	4	16	16	2	0.25	2	≤0.06	0.25
N56738	Ground turkey	4	4	32	16	4	0.12	2	≤0.06	0.12
N58201	Chicken breast	4	2	16	8	2	0.12	2	≤0.06	≤0.06
N60559	Chicken breast	4	4	16	8	1	0.25	1	≤0.06	0.25
N60592	Ground turkey	8	8	32	16	2	0.12	1	≤0.06	0.25
N63148	Ground turkey	8	2	16	8	8	0.12	4	8	8

^a^ CLSI breakpoint.

^b^ NARMS breakpoint.

ATM, aztreonam; CAZ, ceftazidime; CAZ/CLA, ceftazidime–clavulanic acid; CQN, cefquinome; CTX, cefotaxime; CTX/CLA, cefotaxime–clavulanic acid; FEP, cefepime; IPM, imipenem; TZP, piperacillin–tazobactam.

### Conjugation and plasmid typing

**FIG. 1.**
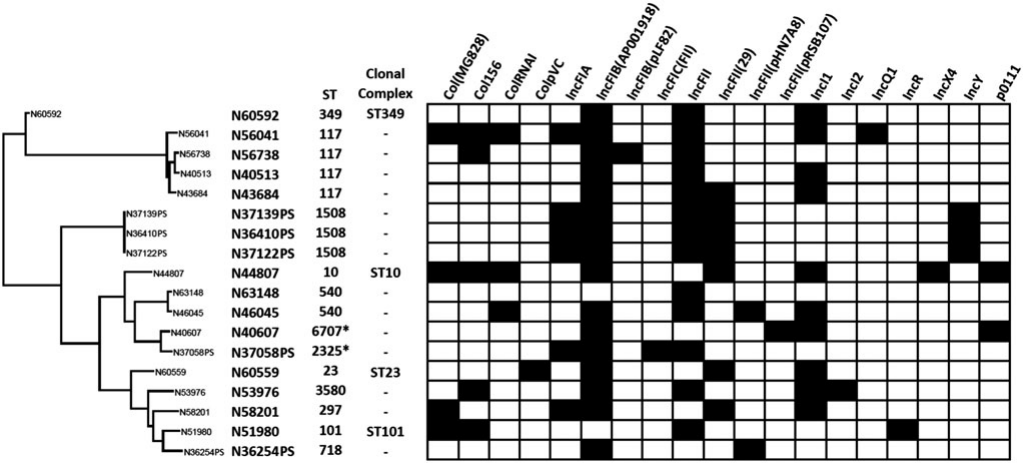
Plasmid and MLST profiling of *bla_CTX-M_*-encoding *Escherichia coli* isolates. Phylogenetic tree generated based on the SNP analysis of the WGS data. The *black* color indicates the presence of the plasmid type based on plasmid finder.^c^ 6707*—a single SNP on recA (T176G) and 2325*—a single SNP on purA (G102A). MLST, multilocus sequence typing; SNP, single nucleotide polymorphism.

At least two plasmid types were detected using PlasmidFinder from each of the *bla*_CTX-M_^+^ *E. coli* isolates (Fig. 1). The conjugation results showed that *bla*_CTX-M_ genes can be transferred by broth mating. The transfer of *bla*_CTX-M_ gene was confirmed by PCR. We further confirmed the plasmid replicon type of the transconjugant with the ESBL phenotype using PCR-based replicon typing. Based on the replicon typing, the two conjugative plasmid types that carried the *CTX-M* genes were IncI1 and IncF, present in isolates from chicken breast and cattle feces, respectively. One of the *bla*_CTX-M_ genes identified from cattle isolates (*bla*_CTX-M-32_) was not transferable by conjugation. Other resistance phenotypes co-transferred by conjugation include Tet^R^ and Smx^R^ (Table 1).

### Resistome analysis in ESBL *E. coli* isolates

**FIG. 2.**
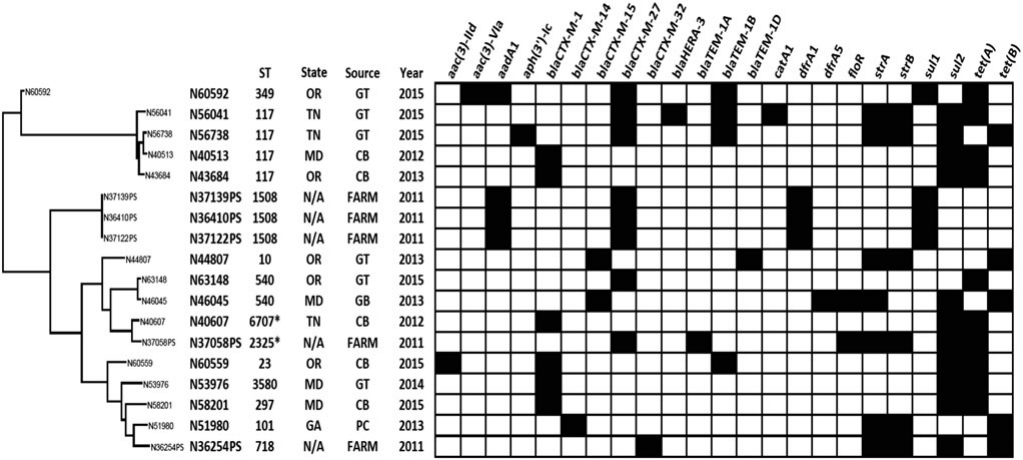
Phylogenetic analysis and summary of MLST profiling and antimicrobial resistance genes identified in phenotypically positive ESBL *E. coli* isolates recovered from cattle and chicken breast. Clustering based on the SNP analysis of WGS data. The *black* color indicates the resistance gene identified. ESBL, extended-spectrum beta-lactamase.

Characterization of resistance genes was conducted using whole-genome sequencing. The distribution of resistance genes is shown in Fig. 2. The ESBL genotypes in our isolates were *bla*_CTX-M-1,_ *bla*_CTX-M-14,_ *bla*_CTX-M-15,_ *bla*_CTX-M-27_, and *bla*_CTX-M-32_. CTX-M β-lactamases exhibit increased hydrolytic activity against cefotaxime and ceftriaxone, but generally not against ceftazidime, which has important implications for laboratory detection. Similarly, in this study, all CTX-M-positive isolates were resistant to cefotaxime and none were resistant to ceftazidime. This is the first report of *bla*_CTX-M-14_- and *bla*_CTX-M-15_-containing *E. coli* isolates from NARMS retail meat program in the United States.

The CTX-M genes identified in our isolates had 100% nucleotide sequence identity with previously reported CTX-M genes. All five of the chicken breast *E. coli* isolates carried *bla*_CTX-M-1_, four of the five cattle isolates (N36410PS, N37058PS, N37122PS, and N37139PS) had *bla*_CTX-M-27_, and one cattle isolate (N36254PS) contained *bla*_CTX-M-32_. Three of the four *bla*_CTX-M-27_^+^ cattle *E. coli* isolates clustered together and had identical MLST type (ST1508) (Fig. 2).

**FIG. 3.**
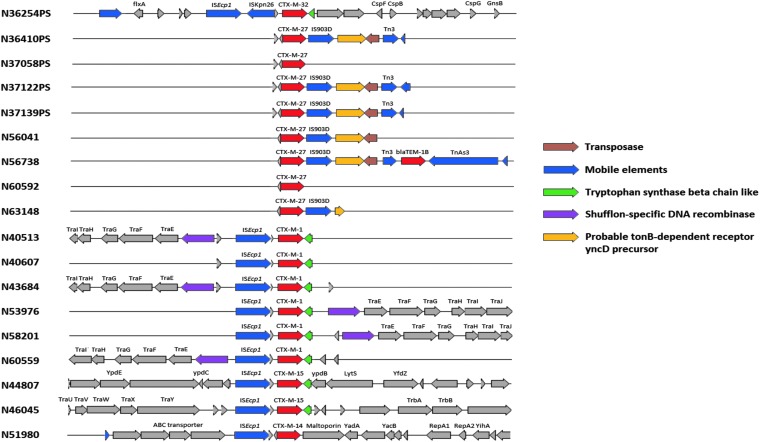
Genetic context of insertion sequence elements (ISEp1, IS903D, and IS26) found in association with *bla_CTX-M_* genes. CTX-M genes are indicated with *red arrows*. Insertion sequences and transposons are represented with *blue arrows*.

*E. coli* isolates recovered from ground turkey carried diverse CTX-M genes, including *bla*_CTX-M-1_ (*n* = 1), *bla*_CTX-M-15_ (*n* = 1), and *bla*_CTX-M-27_ (*n* = 4). A single *E. coli* isolate each from pork chops and ground beef carried *bla*_CTX-M-14_ and *bla*_CTX-M-15_, respectively. The primary mechanisms responsible for the acquisition and mobilization of CTX-M genes are insertions sequences, transposons, and IS*CR1*. In our isolates, we identified IS*Ecp1*, IS*26*, and IS*903-D* mobilization elements (Fig. 3). In chicken breast isolates, *bla*_CTX-M-1_ gene was present as part of an IS*Ecp1* transposition unit and shares sequence similarity with the backbone structure of the IncI plasmid. Conjugation results demonstrated that *tet* and *sul* resistance genes were carried on the same IncI plasmids harboring *bla*_CTX-M-1_ gene.

Streptomycin-resistant isolates (MIC ≥32) carried one or more aminoglycoside resistance genes. The aminoglycoside resistance genes commonly identified in these isolates were *strA* [*aph(3′)-Ib*], *strB* [*aph(6′)-Id*], and *aadA1*. One isolate with a gentamicin MIC >16 μg/mL possessed an *aac(3)-IId* gene. Three *E. coli* isolates (N36410PS, N37122PS, and N37139PS) contained a class 1 integron carrying *aadA1, dfrA*, and *qacE* genes.

Two chloramphenicol-resistant isolates carried the *floR* gene and one contained *catA1*. All, but three *E. coli* isolates were resistant to sulfisoxazole and had either *sul1* or *sul2*. Four also carried the dihydrofolate reductase gene *dfrA*.

## Discussion

CTX-M-producing strains appear to have quickly spread worldwide, with the notable exception of the United States where TEM- and SHV-type ESBL have appeared to predominate until recently. CTX-M ESBLs have been reported in the United States mainly from human clinical isolates of Enterobacteriaceae encoding for CTX-M group 1 and 9.^7,10,16, 25,26^

Infections caused by bacteria producing CTX-M enzymes are not limited to the hospital setting.^27^ Intestinal carriage of CTX-M-producing bacteria in food-producing animals and contamination of retail meat may contribute to increased incidences of infections with ESBL-producing bacteria in humans. Various reports have documented dissemination of ESBL-producing *E. coli* in healthy food-producing animals and animal products in several countries,^12,13,17,28,29^ and the potential of wild birds as possible reservoirs and vehicles for dissemination of CTX-Ms in the United States.^15^

In this study, we are reporting the first *bla*_CTX-M-14_ and *bla*_CTX-M-15_ gene carrying *E. coli* isolate from NARMS retail meat program. All *E. coli* isolates obtained before 2011 from NARMS were phenotypically and genotypically negative for *bla*_CTX-M_.^30^ However, McDermott *et al.* recently identified the first *bla*_CTX-M-1_-positive *Salmonella* isolate recovered from NARMS retail meat samples in the United States.^18^ *Salmonella enterica* serovar Infantis isolates containing *bla*_CTX-M-65_ obtained from chicken, cattle, and human sources collected between 2012 and 2015 in the United States through routine NARMS surveillance have been reported.^19^ Davis *et al.*, reported *bla*_CTX-M_-carrying *E. coli* strains among isolates collected from Washington State cattle in 2011, while none from those collected in 2008.^13^ Investigations of nontyphoidal *Salmonella* isolates of human origin submitted to Center for Disease Control and Prevention (CDC) as part of the NARMS program between 2005 and 2007 identified *Salmonella* isolates producing CTX-M enzymes (*bla*_CTX-M-15_, *bla*_CTX-M-5_, and *bla*_CTX-M-55/57_).^31,32^

The successful spread of CTX-M genes depends on the clonal nature of strains carrying the resistance genes, and mobile genetic elements responsible for its capture and spread. *E. coli* ST131 and ST405 are by far the most important sequence types (STs) associated with the sudden worldwide increase of CTX-M genes, including *bla*_CTX-M-15_^33^ and other CTX-M genes such as *bla*_CTX-M-1_, *bla*_CTX-M-3_, *bla*_CTX-M-10_, and *bla*_CTX-M-14_.^26^ None of our *bla_CTX-M_*^+^ *E. coli* belonged to ST131 or ST405, indicating that the spread of CTX-M genes is not associated with the established clonal strains. We observed diverse STs carrying the same *bla*_CTX-M_ gene, and in some instances, different *bla*_CTX-M_ genes carried by the same ST. For example, *E. coli* isolates recovered from two ground turkey isolates carrying *bla*_CTX-M-27_ and two chicken breast isolates carrying *bla*_CTX-M-1_ were ST117, indicating the potential of the same ST to spread multiple CTX-M genes.

The most commonly reported mobilization elements mediating the spread of CTX-M genes include IS*Ecp1*, IS*CR1*, and IS*26*^34–36^ and phage-related sequences.^37^ In our isolates, different IS elements, including IS*Ecp1*, IS*26*, and IS*903-D* were identified upstream of *bla*_CTX-M-1_, *bla*_CTX-M-27_, and *bla*_CTX-M-32_ genes as previously reported elsewhere.^38–40^ All phenotypically positive ESBL *E. coli* isolates recovered from chicken breast encoded *bla*_CTX-M-1_ as part of an IS*Ecp1* transposition unit and shares sequence similarity with the backbone structure of the IncI plasmid. IncI1 has been shown to be one of the main plasmid lineages that contribute to the dissemination of *bla*_CTX-M-1_ genes in the food chain, including chicken retail meat, the environment, and humans.^41^ In the Netherlands, bacteria producing ESBL isolated from chicken meat and gut of broilers predominantly carried *bla*_CTX-M-1_ located on IncI1 plasmids.^42^ Similarly, Day *et al.* demonstrated a widespread distribution of IncI1 plasmids carrying *bla*_CTX-M-1_ gene among *E. coli* recovered from humans, animals, and food products in Germany, the Netherlands, and the United Kingdom.^43^ Furthermore, a study in the Netherlands revealed the presence of indistinguishable genotypes, CTX-M genes and plasmids, in *E. coli* obtained from poultry, retail chicken meat, and human clinical samples, suggesting possible exchange through food chain.^12^

The *bla*_CTX-M-27_ genes identified in our cattle and ground turkey *E. coli* isolates were associated with IncF plasmids. Horizontal transfer is important in the dissemination of *bla*_CTX-M-27_ gene, as evidenced by the fact that *bla*_CTX-M-27_ genes in our isolates were transferred by conjugation, confirming the location of the gene on a conjugative plasmid. IncF plasmids encode numerous addiction systems that ensure and contribute to the maintenance of antimicrobial resistance determinants and virulence factors even in the absence of antibiotic selection pressure.^44^ IncF replicon-type plasmids carrying *bla*_CTX-M-27_ have been documented in cefotaximase-producing *E. coli* clinical isolates from Dublin, Ireland.^45^ Plasmids carrying *bla*_CTX-M_ genes are often self-conjugative and carry additional resistance determinants,^46^ greatly facilitating widespread distribution of alleles in different environments. A recent report has documented a case of ceftriaxone treatment failure caused by *Salmonella* Typhimurium due to the *in vivo* acquisition of a *bla*_CTX-M-27_-encoding IncFII group transmissible plasmid.^47^

The presence of *bla*_CTX-M-15_ and *bla*_CTX-M-14_ was reported in most U.S. medical centers participating in the Meropenem Yearly Susceptibility Test Information Collection (MYSTIC) program in 2007.^10,25^ In this study, we are reporting for the first time *bla*_CTX-M-15_- and *bla*_CTX-M-14_-encoding *E. coli* from retail meat samples collected in 2013. CTX-M-15 and CTX-M-14, the two most frequently identified CTX-M enzyme worldwide, have been detected in bacteria isolated from humans, animals, and the environment.^17,42,48^ A recent study from six community hospitals in North Carolina and Virginia from 2010 to 2012 demonstrated that 80% of ESBL-producing isolates contained CTX-M enzymes. In these isolates, ST131 was associated with 48% of *bla*_CTX-M-15_-producing *E. coli* isolates and 66% of the *bla*_CTX-M-14_-producing *E. coli* isolates.^49^ While the prevalence of these two successful CTX-M enzymes is low from domestic food animal sources, monitoring will continue to help determine whether this mechanism is becoming more widespread among animal and food strains of *E. coli* in the United States.

## Notes

^a^ https://blast.ncbi.nlm.nih.gov/

^b^ http://snp-pipeline.readthedocs.io/

^c^ https://cge.cbs.dtu.dk/services/PlasmidFinder

^d^ https://pubmlst.org/

^c^ https://cge.cbs.dtu.dk/services/PlasmidFinder

## References

[1] Bradford P.A. 2001 Extended-spectrum beta-lactamases in the 21st century: characterization, epidemiology, and detection of this important resistance threat. Clin. Microbiol. Rev. 14:933–951 11585791 10.1128/CMR.14.4.933-951.2001 PMC89009

[2] Paterson D.L., and Bonomo R.A. 2005 Extended-spectrum beta-lactamases: a clinical update. Clin. Microbiol. Rev. 18:657–686 16223952 10.1128/CMR.18.4.657-686.2005 PMC1265908

[3] Shah P.M., and Stille W. 1983 *Escherichia coli* and *Klebsiella pneumoniae* strains more susceptible to cefoxitin than to third generation cephalosporins. J. Antimicrob. Chemother. 11:597–598 6350256 10.1093/jac/11.6.597

[4] CDC.2013 Antibiotic Resistance Threats in the United States, 2013. Centers for Disease Control and Prevention Atlanta, GA Available at www.cdc.gov/drugresistance/pdf/ar-threats-2013-508.pdf

[5] Woodford N., Carattoli A., Karisik E., Underwood A., Ellington M.J., and Livermore D.M. 2009 Complete nucleotide sequences of plasmids pEK204, pEK499, and pEK516, encoding CTX-M enzymes in three major *Escherichia coli* lineages from the United Kingdom, all belonging to the international O25:H4-ST131 clone. Antimicrob. Agents Chemother. 53:4472–4482 19687243 10.1128/AAC.00688-09 PMC2764225

[6] Pitout J.D., Nordmann P., Laupland K.B., and Poirel L. 2005 Emergence of Enterobacteriaceae producing extended-spectrum beta-lactamases (ESBLs) in the community. J. Antimicrob. Chemother. 56:52–59 15917288 10.1093/jac/dki166

[7] Lewis J.S., Herrera M., Wickes B., Patterson J.E., and Jorgensen J.H. 2007 First report of the emergence of CTX-M-type extended-spectrum beta-lactamases ( ESBLs) as the predominant ESBL isolated in a U.S. health care system. Antimicrob. Agents Chemother. 51:4015–4021 17724160 10.1128/AAC.00576-07 PMC2151438

[8] Canton R., Novais A., Valverde A., Machado E., Peixe L., Baquero F., and Coque T.M. 2008 Prevalence and spread of extended-spectrum beta-lactamase-producing Enterobacteriaceae in Europe. Clin. Microbiol. Infect. 14 (Suppl 1): 144–153 18154538 10.1111/j.1469-0691.2007.01850.x

[9] D'Andrea M.M., Arena F., Pallecchi L., and Rossolini G.M. 2013 CTX-M-type beta-lactamases: a successful story of antibiotic resistance. Int. J. Med. Microbiol. 303:305–317 23490927 10.1016/j.ijmm.2013.02.008

[10] Castanheira M., Mendes R.E., Rhomberg P.R., and Jones R.N. 2008 Rapid emergence of blaCTX-M among Enterobacteriaceae in Medical Centers U.S.: molecular evaluation from the MYSTIC Program ( 2007). Microb. Drug Resist. 14:211–216 18707552 10.1089/mdr.2008.0827

[11] Wittum T.E., Mollenkopf D.F., Daniels J.B., Parkinson A.E., Mathews J.L., Fry P.R., Abley M.J., and Gebreyes W.A. 2010 CTX-M-type extended-spectrum beta-lactamases present in *Escherichia coli* from the feces of cattle in Ohio, United States. Foodborne Pathog. Dis. 7:1575–1579 10.1089/fpd.2010.0615 20707724

[12] Leverstein-van Hall M.A., Dierikx C.M., Cohen S.J., Voets G.M., van den Munckhof M.P., van Essen-Zandbergen A., Platteel T., Fluit A.C., Sande-Bruinsma N., Scharinga J., Bonten M.J., Mevius D.J.; National ESBL Surveillance Group 2011 Dutch patients, retail chicken meat and poultry share the same ESBL genes, plasmids and strains. Clin. Microbiol. Infect. 17:873–880 21463397 10.1111/j.1469-0691.2011.03497.x

[13] Davis M.A., Sischo W.M., Jones L.P., Moore D.A., Ahmed S., Short D.M., and Besser T.E. 2015 Recent emergence of *Escherichia coli* with cephalosporin resistance conferred by blaCTX-M on Washington State dairy farms. Appl. Environ. Microbiol. 81:4403–4410 25911480 10.1128/AEM.00463-15 PMC4475894

[14] Carattoli A., Lovari S., Franco A., Cordaro G., Di Matteo P., and Battisti A. 2005 Extended-spectrum beta-lactamases in *Escherichia coli* isolated from dogs and cats in Rome, Italy, from 2001 to 2003. Antimicrob. Agents Chemother. 49:833–835 15673782 10.1128/AAC.49.2.833-835.2005 PMC547336

[15] Poirel L., Potron A., De La Cuesta C., Cleary T., Nordmann P., and Munoz-Price L.S. 2012 Wild coastline birds as reservoirs of broad-spectrum-beta-lactamase-producing Enterobacteriaceae in Miami Beach, Florida. Antimicrob. Agents Chemother. 56:2756–2758 22314536 10.1128/AAC.05982-11 PMC3346599

[16] Moland E.S., Black J.A., Hossain A., Hanson N.D., Thomson K.S., and Pottumarthy S. 2003 Discovery of CTX-M-like extended-spectrum beta-lactamases in *Escherichia coli* isolates from five US States. Antimicrob. Agents Chemother. 47:2382–2383 12821506 10.1128/AAC.47.7.2382-2383.2003 PMC161829

[17] Cottell J.L., Webber M.A., Coldham N.G., Taylor D.L., Cerdeno-Tarraga A.M., Hauser H., Thomson N.R., Woodward M.J., and Piddock L.J. 2011 Complete sequence and molecular epidemiology of IncK epidemic plasmid encoding blaCTX-M-14. Emerg. Infect. Dis. 17:645–652 21470454 10.3201/eid1704.101009 PMC3377399

[18] McDermott P.F., Tyson G.H., Kabera C., Chen Y., Li C., Folster J.P., Ayers S.L., Lam C., Tate H.P., and Zhao S. 2016 Whole-genome sequencing for detecting antimicrobial resistance in nontyphoidal *Salmonella*. Antimicrob. Agents Chemother. 60:5515–5520 27381390 10.1128/AAC.01030-16 PMC4997858

[19] Tate H., Folster J.P., Hsu C.H., Chen J., Hoffmann M., Li C., Morales C., Tyson G.H., Mukherjee S., Brown A.C., Green A., Wilson W., Dessai U., Abbott J., Joseph L., Haro J., Ayers S., McDermott P.F., and Zhao S. 2017 Comparative analysis of extended-spectrum-beta-lactamase CTX-M-65-producing *Salmonella enterica* serovar Infantis isolates from humans, food animals, and retail chickens in the United States. Antimicrob. Agents Chemother. 61:e00488-17 28483962 10.1128/AAC.00488-17 PMC5487606

[20] CLSI. 2017 Performance Standards for Antimicrobial Susceptibility Testing; 27th Informational Supplement. CLSI Document M100–S27. Clinical and Laboratory Standards Institute, Wayne, PA

[21] Johnson T.J., Wannemuehler Y.M., Johnson S.J., Logue C.M., White D.G., Doetkott C., and Nolan L.K. 2007 Plasmid replicon typing of commensal and pathogenic *Escherichia coli* isolates. Appl. Environ. Microbiol. 73:1976–1983 17277222 10.1128/AEM.02171-06 PMC1828809

[22] Zankari E., Hasman H., Cosentino S., Vestergaard M., Rasmussen S., Lund O., Aarestrup F.M., and Larsen M.V. 2012 Identification of acquired antimicrobial resistance genes. J. Antimicrob. Chemother. 67:2640–2644 22782487 10.1093/jac/dks261 PMC3468078

[23] Lee T.H., Guo H., Wang X., Kim C., and Paterson A.H. 2014 SNPhylo: a pipeline to construct a phylogenetic tree from huge SNP data. BMC Genomics 15:162 24571581 10.1186/1471-2164-15-162 PMC3945939

[24] Inouye M., Dashnow H., Raven L.A., Schultz M.B., Pope B.J., Tomita T., Zobel J., and Holt K.E. 2014 SRST2: rapid genomic surveillance for public health and hospital microbiology labs. Genome Med. 6:90 25422674 10.1186/s13073-014-0090-6 PMC4237778

[25] Castanheira M., Sader H.S., and Jones R.N. 2010 Antimicrobial susceptibility patterns of KPC-producing or CTX-M-producing Enterobacteriaceae. Microb. Drug Resist. 16:61–65 20192819 10.1089/mdr.2009.0031

[26] Naseer U., and Sundsfjord A. 2011 The CTX-M conundrum: dissemination of plasmids and *Escherichia coli* clones. Microb. Drug Resist. 17:83–97 21281129 10.1089/mdr.2010.0132

[27] Pitout J.D., and Laupland K.B. 2008 Extended-spectrum beta-lactamase-producing Enterobacteriaceae: an emerging public-health concern. Lancet Infect. Dis. 8:159–166 18291338 10.1016/S1473-3099(08)70041-0

[28] Horton R.A., Randall L.P., Snary E.L., Cockrem H., Lotz S., Wearing H., Duncan D., Rabie A., McLaren I., Watson E., La Ragione R.M., and Coldham N.G. 2011 Fecal carriage and shedding density of CTX-M extended-spectrum {beta}-lactamase-producing *Escherichia coli* in cattle, chickens, and pigs: implications for environmental contamination and food production. Appl. Environ. Microbiol. 77:3715–3719 21478314 10.1128/AEM.02831-10 PMC3127594

[29] Tamang M.D., Nam H.M., Kim T.S., Jang G.C., Jung S.C., and Lim S.K. 2011 Emergence of extended-spectrum beta-lactamase (CTX-M-15 and CTX-M-14)-producing nontyphoid *Salmonella* with reduced susceptibility to ciprofloxacin among food animals and humans in Korea. J. Clin. Microbiol. 49:2671–2675 21613434 10.1128/JCM.00754-11 PMC3147890

[30] Zhao S., Blickenstaff K., Glenn A., Ayers S.L., Friedman S.L., Abbott J.W., and McDermott P.F. 2009 Beta-lactam resistance in *Salmonella* strains isolated from retail meats in the United States by the National Antimicrobial Resistance Monitoring System between 2002 and 2006. Appl. Environ. Microbiol. 75:7624–7630 19854922 10.1128/AEM.01158-09 PMC2794113

[31] Sjolund-Karlsson M., Howie R., Krueger A., Rickert R., Pecic G., Lupoli K., Folster J.P., and Whichard J.M. 2011 CTX-M-producing non-Typhi *Salmonella* spp. isolated from humans, United States. Emerg. Infect. Dis. 17:97–99 21192864 10.3201/eid1701.100511 PMC3204627

[32] Sjolund-Karlsson M., Rickert R., Matar C., Pecic G., Howie R.L., Joyce K., Medalla F., Barzilay E.J., and Whichard J.M. 2010 *Salmonella* isolates with decreased susceptibility to extended-spectrum cephalosporins in the United States. Foodborne Pathog. Dis. 7:1503–1509 20704496 10.1089/fpd.2010.0607

[33] Peirano G., and Pitout J.D. 2010 Molecular epidemiology of *Escherichia coli* producing CTX-M beta-lactamases: the worldwide emergence of clone ST131 O25:H4. Int. J. Antimicrob. Agents 35:316–321 20060273 10.1016/j.ijantimicag.2009.11.003

[34] Toleman M.A., Bennett P.M., and Walsh T.R. 2006 ISCR elements: novel gene-capturing systems of the 21st century? Microbiol. Mol. Biol. Rev. 70:296–316 10.1128/MMBR.00048-05 PMC1489542 16760305

[35] Cullik A., Pfeifer Y., Prager R., von Baum H., and Witte W. 2010 A novel IS26 structure surrounds blaCTX-M genes in different plasmids from German clinical *Escherichia coli* isolates. J. Med. Microbiol. 59:580–587 20093380 10.1099/jmm.0.016188-0

[36] Partridge S.R., Ellem J.A., Tetu S.G., Zong Z., Paulsen I.T., and Iredell J.R. 2011 Complete sequence of pJIE143, a pir-type plasmid carrying ISEcp1-blaCTX-M-15 from an *Escherichia coli* ST131 isolate. Antimicrob. Agents Chemother. 55:5933–5935 21911569 10.1128/AAC.00639-11 PMC3232798

[37] Oliver A., Coque T.M., Alonso D., Valverde A., Baquero F., and Canton R. 2005 CTX-M-10 linked to a phage-related element is widely disseminated among Enterobacteriaceae in a Spanish hospital. Antimicrob. Agents Chemother. 49:1567–1571 15793141 10.1128/AAC.49.4.1567-1571.2005 PMC1068625

[38] Bou G., Cartelle M., Tomas M., Canle D., Molina F., Moure R., Eiros J.M., and Guerrero A. 2002 Identification and broad dissemination of the CTX-M-14 beta-lactamase in different *Escherichia coli* strains in the northwest area of Spain. J. Clin. Microbiol. 40:4030–4036 10.1128/JCM.40.11.4030-4036.2002 PMC139670 12409370

[39] Chanawong A., M'Zali F.H., Heritage J., Xiong J.H., and Hawkey P.M. 2002 Three cefotaximases, CTX-M-9, CTX-M-13, and CTX-M-14, among Enterobacteriaceae in the People's Republic of China. Antimicrob. Agents Chemother. 46:630–637 11850241 10.1128/AAC.46.3.630-637.2002 PMC127467

[40] Dutour C., Bonnet R., Marchandin H., Boyer M., Chanal C., Sirot D., and Sirot J. 2002 CTX-M-1, CTX-M-3, and CTX-M-14 beta-lactamases from Enterobacteriaceae isolated in France. Antimicrob. Agents Chemother. 46:534–537 11796372 10.1128/AAC.46.2.534-537.2002 PMC127047

[41] Zurfluh K., Jakobi G., Stephan R., Hachler H., and Nuesch-Inderbinen M. 2014 Replicon typing of plasmids carrying bla CTX-M-1 in Enterobacteriaceae of animal, environmental and human origin. Front. Microbiol. 5:555 25400623 10.3389/fmicb.2014.00555 PMC4214192

[42] Fischer E.A., Dierikx C.M., van Essen-Zandbergen A., van Roermund H.J., Mevius D.J., Stegeman A., and Klinkenberg D. 2014 The IncI1 plasmid carrying the blaCTX-M-1 gene persists in in vitro culture of a *Escherichia coli* strain from broilers. BMC Microbiol. 14:77 24666793 10.1186/1471-2180-14-77 PMC3987674

[43] Day M.J., Rodriguez I., van Essen-Zandbergen A., Dierikx C., Kadlec K., Schink A.K., Wu G., Chattaway M.A., DoNascimento V., Wain J., Helmuth R., Guerra B., Schwarz S., Threlfall J., Woodward M.J., Coldham N., Mevius D., and Woodford N. 2016 Diversity of STs, plasmids and ESBL genes among *Escherichia coli* from humans, animals and food in Germany, the Netherlands and the UK. J. Antimicrob. Chemother. 71:1178–1182 10.1093/jac/dkv485 26803720

[44] Carattoli A. 2011 Plasmids in Gram negatives: molecular typing of resistance plasmids. Int. J. Med. Microbiol. 301:654–658 21992746 10.1016/j.ijmm.2011.09.003

[45] Burke L., Humphreys H., and Fitzgerald-Hughes D. 2016 The molecular epidemiology of resistance in cefotaximase-producing *Escherichia coli* clinical isolates from Dublin, Ireland. Microb. Drug Resist. 22:552–558 27003161 10.1089/mdr.2015.0154

[46] Rossolini G.M., D'Andrea M.M., and Mugnaioli C. 2008 The spread of CTX-M-type extended-spectrum beta-lactamases. Clin. Microbiol. Infect. 14 (Suppl 1): 33–41 18154526 10.1111/j.1469-0691.2007.01867.x

[47] McCollister B., Kotter C.V., Frank D.N., Washburn T., and Jobling M.G. 2016 Whole-genome sequencing identifies in vivo acquisition of a blaCTX-M-27-carrying IncFII transmissible plasmid as the cause of ceftriaxone treatment failure for an invasive *Salmonella enterica* serovar Typhimurium infection. Antimicrob. Agents Chemother. 60:7224–7235 27671066 10.1128/AAC.01649-16 PMC5119002

[48] Hawkey P.M., and Jones A.M. 2009 The changing epidemiology of resistance. J. Antimicrob. Chemother. 64 (Suppl 1): i3–i10 19675017 10.1093/jac/dkp256

[49] Chen L.F., Freeman J.T., Nicholson B., Keiger A., Lancaster S., Joyce M., Woods C.W., Cook E., Adcock L., Louis S., Cromer A.L., Sexton D.J., and Anderson D.J. 2014 Widespread dissemination of CTX-M-15 genotype extended-spectrum-beta-lactamase-producing Enterobacteriaceae among patients presenting to community hospitals in the southeastern United States. Antimicrob. Agents Chemother. 58:1200–1202 24247126 10.1128/AAC.01099-13 PMC3910860

